# The effect of age and perturbation time on online control during rapid pointing

**DOI:** 10.1371/journal.pone.0222219

**Published:** 2019-09-12

**Authors:** Jessica L. O’Rielly, Anna Ma-Wyatt

**Affiliations:** School of Psychology, University of Adelaide, Adelaide, South Australia, Australia; Tokyo Daigaku, JAPAN

## Abstract

Visual and proprioceptive information is used differently at different phases of a reach. The time at which a target perturbation occurs during a reach therefore has a significant impact on how an individual can compensate for this perturbation though online control. With healthy ageing, there are notable changes to both sensory and motor control that impact motor performance. However, how the online control process changes with age is not yet fully understood. We used a target perturbation paradigm and manipulated the time at which a target perturbation occurred during the reach to investigate how healthy ageing impacts sensorimotor control. We measured how the latency of the correction and the magnitude of the corrective response changed with perturbation time and quantified the difference across groups using a percentage difference measure. For both groups, online corrections to early perturbations were more easily accounted for than those to late perturbations, despite late perturbations eliciting faster correction latencies. While there was no group difference in accuracy, older participants were slower overall and produced a correction to a change in target location proportionally less often despite similar correction latencies. We speculate that the differences in the time during the reach that the correction is first identified may explain the differences in correction latencies observed between the perturbation time conditions.

## Introduction

Eye-hand coordination is a prominent part of everyday activity. Actions as simple as picking up an apple to the complexities of computer interaction all require accurate and precise hand movements that need to be coordinated using both visual and proprioceptive information. One aspect of eye-hand coordination that is particularly relevant to the fluid and flexible control of action is the online control of movement. This relates to the ability to modify ongoing action inflight to achieve the task goal. The online control of movement has been widely investigated in younger adults using the double-step paradigm. In this paradigm, the reaching target is displaced on or close to reach onset and the participant is required to adapt their movement while the hand is still in flight to account for the location change. Performance in this paradigm is quantified in terms of how quickly and accurately the individual is able to complete this correction (e.g. [1–3]).

Models of goal directed reaching suggest that targeted reaching movements require several processing steps [4]. The target is first localised, motor plans generated, and finally motor commands are sent to the arm muscles, resulting in a movement. During the movement process a forward model of the dynamics of the arm is generated. This model receives the sensory inflow and a copy of the motor outflow as inputs and generates an estimate of the movement endpoint location as output. Visual and proprioceptive feedback is used to compare this estimate with information about the target location. The difference between actual and predicted sensory feedback is the sensory error and in the case of discrepancy, an error signal is produced that triggers a modification of the ongoing motor command [4]. Through this online control process, sensory feedback and feedforward information can be used during the reach to influence the outcome [5]. While online control, and models conceptualising this process, have been thoroughly investigated within younger adults, there is limited evidence about how healthy ageing affects online updating. Indeed, there is currently no formal account for how the multiple visual and motor processes impacted by ageing (see [6] for a full review) impact the sensorimotor control process. One of the first steps in developing a model of sensorimotor control that can predict performance across ages is to provide quantitative data about the effect of ageing on online control for a controlled parameter space. As such, the aim of the current experiment is to provide a parsimonious account of the effect of age on online control throughout the reaching process.

Much of the work investigating online control in younger participants has focused on tasks where target perturbations occur on or close to movement initiation. These studies have shown that in response to these target perturbations, the hand path is smoothly corrected towards the location of the displaced target [2], an observation in line with multiple models of motor control (e.g. [7–9]). There is limited evidence however, to inform how this process changes with online control and healthy ageing. Sarlegna [10] compared performance of younger and older participants in a double-step reaching task to virtual targets that were perturbed at reach onset. He demonstrated that older participants took significantly longer to initiate a corrective movement and produced corrections that were proportionally less complete than the younger controls, concluding that ageing significantly affected the online control of movement. However, Sarlegna [10] asked participants to control only the direction of the corrective response as participants were required perform a reach by fully extending their arm, in order to pass through the virtual targets rather than stop at them. This decreases the accuracy requirements of the movement, as only direction and not amplitude or termination need to be controlled. Assessing endpoint accuracy explicitly would have been a beneficial measure of the functional output of the sensorimotor system during online control as previous research has indicated that older subjects actively slow their responses to preserve accuracy in an attempt to compensate for slower processing speed [11]. Kimura, Kadota and Kinoshita [12] similarly found an age-related functional deficit in online control. They showed that the variance in the directional error of the corrective response correlated with the variance of the reaching trajectory at the half way point for younger participants but did not for older participants. They suggested that the corrective response may play a role in decreasing the endpoint variability of the reach. If this corrective process is indeed impaired with age as Sarlegna [10] and Kimura, Kadota and Kinoshita [12] suggest, then this may help explain some of the variability seen in the pointing performance of older participants.

Rossit and Harvey [13] offered an alternative explanation to the apparent age-related deficit associated with the online control of movement. They used a double-step paradigm to compare online control across younger and older participants. They demonstrated that although older subjects took significantly longer to initiate, execute and correct a goal directed reach, they performed with a level of accuracy comparable to that of the younger participants. Since this slowing was also observed on non-perturbed trials, Rossit and Harvey [13] suggested that ageing does not specifically affect an individual’s ability to perform corrections to goal directed reaches but produces a general slowing and increased variability of movement planning, initiation and execution. However, Rossit and Harvey [13] also noted that their classification of a successful correction did not strictly fit the criteria of an automatic, fast correction, as the correction latency reported was significantly longer than that previously suggested (e.g. 110-150ms; [14,15]). This longer duration would also provide more time for integration of new visual feedback gained from the perturbed location of the target to improve endpoint accuracy (e.g. [16]) and may explain the comparable accuracy of young and older participants in their results.

Indeed, previous work has demonstrated that the timing of the perturbation and the subsequent time available to complete this correction limits the final accuracy of the movement. While, there is limited evidence detailing the effects of healthy ageing on online updating, particularly throughout the reaching process, some insight may be gained from understanding the performance of healthy, younger adults tested under conditions designed to manipulate the timing of the target perturbation. Komilis, Pélisson and Prablanc [17] conducted a double-step reaching task where the perturbation occurred at either reach onset or peak velocity. They demonstrated that when the target was perturbed at movement onset participants compensated for 88–100% of the target displacement. In contrast, when the target was displaced at peak velocity participants were unable to fully account for the change in target position and compensated for only 20–40% of the perturbation. Liu and Todorov [2] similarly found incomplete corrections to target perturbations that occurred considerably after reach onset. They hypothesized that later target perturbations are less effectively compensated for than those that occur earlier in the reach due to a potential trade-off between endpoint accuracy and the stopping stability requirements at the end of the movement. Liu and Todorov [2] suggest that achieving stability in stopping a reaching movement may potentially compromise the sensorimotor system’s ability to respond to positional errors at the end of the reach. This is interesting because, as mentioned above, studies investigating age related performance in online control have largely focused on perturbations that occur early in the reach which are perhaps more easily accounted for than those which occur later. These findings would be in line with O’Rielly and Ma-Wyatt [18] who also found incomplete corrections to perturbations that occurred later in the reach despite an increase in reach duration.

These results demonstrate that the time at which a target perturbation occurs during the reach has a significant effect on how an individual can compensate for this perturbation, but it is not yet understood how healthy ageing may impact this online correction throughout the duration of the reach. We used a target perturbation paradigm and manipulated the time at which the target perturbation occurred after movement onset to gain insight into how ageing affects online control throughout the movement process. Participants completed a perturbation task in which perturbations were presented early in the reach (0ms after reach onset) and at an intermediate time in the reach (200ms after reach onset), with a reach duration time pressure of 550ms. These times were selected to determine how each group was able to use the available time to correct their reach when a perturbation occurred at reach onset and part way into the reach. We did this to quantify how quickly a reaching movement can be corrected and how the errors associated with a target perturbation changed across the time in the reach it occurred and across age groups.

Given the well documented heterogeneous nature of movement times of older participants, it is difficult to define target perturbation times that occur at equivalent times in the movement for all participants. In an attempt to systematically manipulate perturbation time across participants we chose to initiate the target perturbation at fixed intervals post reach initiation and provide feedback based on the overall duration of the movement. This approach has been used extensively with younger participants [19] and has been shown to reduce this variability in movement times.

There is evidence to suggest that older participants have longer deceleration times and lower peak velocities compared to younger participants and this is expected for our older participants regardless of perturbation times [20]. To quantify the effect of perturbation time on online control, we used an analysis of the latency and accuracy of the reach trajectory correction. Older participants are expected to show an increased sensorimotor correction latency, maximum path offset and the latency of the maximum path offset as defined by changes to the reaching trajectory in response to a target perturbation (see methods section for a full description of these measures). Kinematic markers such as correction latency, reach velocity and deceleration time are sensitive to changes in reach correction and were used to quantify performance across perturbation conditions. These measures will provide insight into how sensory information is incorporated into the ongoing movement trajectory at different times during the reach. Performance measures such as accuracy and reach duration provide insight into the overall integrity of the sensorimotor system in terms of the outcome of the reach for different perturbation time conditions. We expect older participants to be slower in their reach latency and duration across all perturbation conditions but maintain a degree of reach accuracy comparable to that of younger participants.

## Method

### Participants

Younger and older participants were recruited from within and around the School of Psychology at the University of Adelaide through the use of recruitment flyers and posters. 16 (8 females) older participants, mean age 70 years (62–78) and 16 (10 females) younger participants, mean age 26 years (20–30) were tested. All participants were naïve to the purposes of the experiment and identified no previous history of neurological, physical or ocular abnormality. Participants’ travel expenditure was compensated up to an amount of $AU15. This study was approved by The School of Psychology’s Human Ethics Committee at the University of Adelaide and was conducted according to the principles expressed in the Declaration of Helsinki. All participants gave written, informed consent and were free to withdraw at any time without penalty.

All participants completed a series of screening measures to ensure normal cognitive and physical functioning relative to age. Cognitive functioning was assessed with the Mini Mental state Exam [21]. Physical ability was assessed with the Activities of daily living scale [22]. Contrast sensitivity was measured with the Pelli-Robson Contrast Sensitivity Chart [23]. Visual acuity was determined using a Snellen visual acuity chart [24]. Stereo ability was determined using the Randot Stereo test [25]. The standard protocols according to manufacturers' guidelines were followed for all of these tests, all of which were measured binocularly. Age related norms for each task were used to assess deviance from normality. No participant scores were outside of normal age-related functioning. Handedness was assessed with the Edinburgh Handedness Inventory [26]. Two older participants and four younger participants were identified as left handed.

### Apparatus

The experimental apparatus configuration was in line with that described within O’Rielly and Ma-Wyatt [18]. Briefly, a system unit with an Intel(R) i7 core and processor speed of 3.07 GHz was used to execute program functions. A LCD 17-inch ELO touchscreen monitor overlaid with a touch sensitive layer was used to display the stimuli and collect the touch responses. A Polhemus Liberty electromagnetic motion tracking system at a sampling rate of 240 Hz was used to record the reach trajectory. The Polhemus sensor was attached to the forefinger of the participant’s dominant hand with a custom-built Velcro glove. Custom software to run the experiment was written in MATLAB (Mathworks), using the Psychophysics Toolbox [27, 28].

### Task procedure

The experiment was conducted in a slightly darkened room. Participants were seated at a comfortable reaching distance (40cm) from the monitor and allowed time to acclimatise to the lowered levels of light. Before completing a block of experimental trials participants completed 10–20 practice trials, until they reported being familiar with the procedure.

**Fig 1:**
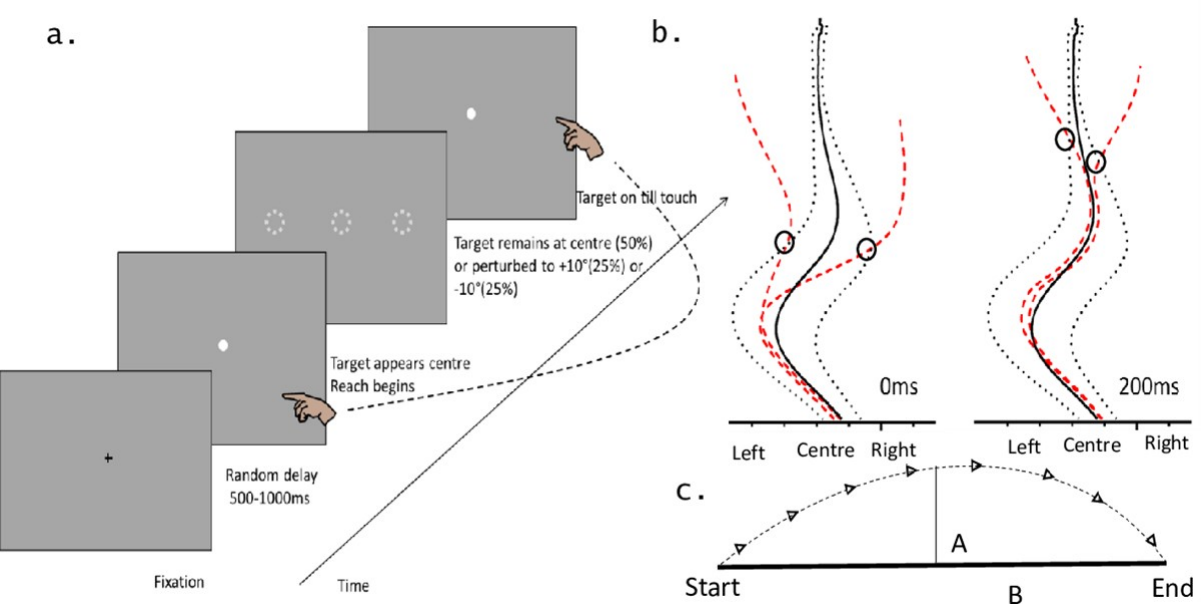
Experimental design and measures. (a) Diagram of a single trial. Participants fixate on fixation cross. After a random delay the initial target appears. The participant begins their reach and the target either remains stationary (50% of trials) or is perturbed to 10° eccentricity left (25% of trials) or right (25% of trials) of the initial target at such time designated by the perturbation time condition (0ms or 200ms) post reach initiation. Target remains visible until touch. (b) The identification of the latency of the corrective movements. Trajectories towards the non-perturbed target position in a single block were averaged for each participant (solid black line) and ±1.5 standard deviations around each average trajectory was calculated (dashed black line). The point in time when the trajectory describing a single target perturbation trial (dashed red line) exceeded the boundary designated by the 1.5SD was defined as the onset of corrective movement (indicated by a circle on Fig 2). Data from a single younger exemplar depicting a left and right perturbation trial is presented. (c) Atkeson and Hollerbach’s [29] measure of path linearity (L) can be calculated as L = A/B where A is the maximum normal distance from the path (dotted line, L) to a straight-line joining start and end points and B is this straight-line distance.

A diagram of the task procedure is presented in Fig 1A. The general double-step experimental procedure used has been described previously [18]. In this study, we altered the paradigm to explicitly quantify the latency of the corrective movement across conditions and age groups. At the beginning of each trial a black fixation cross was presented in the centre of the computer screen that maintained a stable grey background, luminance of 44 cd/m^2^. Participants were instructed to fixate on this cross while holding down the mouse key with their dominant hand. We used a variable delay of 500-1000ms, generated by a random permutation of numbers within this range, to circumvent anticipatory error. After this delay, the cross was extinguished and the target presented. The participant had been instructed to make a reach towards the screen as soon as they saw the target. The target was a high contrast (60%) white dot that subtended 0.5° in diameter at a viewing distance of 40cm. When the participant’s finger made contact with the monitor, the trial ended, and the participant returned their finger to the experimental set-up position in preparation for the next trial.

The target initially appeared at a central location 4.5° above the fixation point. Participants initiated a reaching movement and after a variable amount of time, it either remained stationary (50% of trials) or disappeared and reappeared at a second location. On these perturbation trials, the target was shifted laterally to the right (25% of trials) or left (25% of trials) 10° from centre. Target perturbation trials were randomly interleaved with static trials. The target remained visible at the final location until touch. The time of perturbation was relative to the participant’s initiation of a pointing trial by the release of the mouse button and could occur at either 0ms or 200ms post release. These times were selected to determine how each group was able to use the available time to correct their reach when a perturbation occurred at reach onset and part way into the reach. Participants were provided feedback regarding their reach duration but not accuracy. Feedback limits were informed by previous work within our lab [18]. We did this in an effort to introduce consistent timing which allowed us to investigate changes in reaching error associated with the target perturbation depending on when in the reach the perturbation occurred, either 0ms or 200ms, and quantify any changes in reaching error associated.

Testing was conducted over the course of a single day, with no testing session lasting longer than one hour. Each block within the testing session took about 5 minutes to complete and consisted of 80 trials. Regular breaks within a session were used to prevent participant fatigue. Since it was a repeated measures, within subjects design, participants completed all experimental conditions across 6 blocks of trials. This provided a total of 480 trials per participant. Participant gaze position information was also recorded throughout the trial however analysis relating to this will be reported elsewhere.

## Data analysis

Custom analysis software was developed in MATLAB (Mathworks). Data was initially visually inspected. Trials in which there was an apparatus recording error or a participant error (e.g. the touch screen failed to register a touch response, or the participant initiated a reach response before the target appearance) were removed from analysis. This resulted in 6% of trials being removed from analysis within the younger group and 9% of trials within the older group. Individual participant data was then processed according to perturbation time and direction, performance outliers were detected and removed using the median absolute deviation (MAD) method [30]; This resulted in an additional 4% of trials being removed from the younger group and 10% of trials from the older group. This overall process resulted in a disproportionate number of trials removed across participants, as such participant mean scores for each condition were used for analysis of dependent variables.

### Dependent variables

As the online control of a goal directed reach is both a temporal and spatial problem, performance was assessed across a number of spatial and temporally relevant measures. Reach latency was calculated as the time between the stimulus presentation and the reach initiation as defined by the release of the mouse key. The reach duration was taken from the release of the mouse key until a touch response was recorded. Reach accuracy was calculated by comparing the difference in degrees of visual angle on the horizontal plane between the touch location, recorded on the touch screen, and the target location. We used an absolute value for our analysis since the values capturing a directional effect, which could be both positive and negative, may hide a significant effect when averaged. This method has been used previously (e.g. [13, 18]).

All kinematic data were low-pass filtered at 20 Hz with a two way 4th order Butterworth filter. Peak velocity was defined as the maximum velocity for any given trial in centimetres per second. Acceleration time was defined as the duration between movement initiation (the release of the mouse button) and peak velocity and deceleration time the duration between peak velocity and touch response (defined by the touch screen), both captured in milliseconds.

### Correction latency

In line with previous work [13, 19] the latency of the correction response to a target perturbation was determined by the comparison of the horizontal trace position of the finger during individual target perturbation trials to the horizontal trace position of the averaged non-perturbation trials. Fig 1B presents a diagram of this process. Firstly, for each participant non-perturbed horizontal reach vectors were averaged and ±1.5 standard deviations around the average trajectory were calculated. Single target perturbation trials were then individually compared against that participant’s average horizontal trajectory. In line with previous work estimating the minimum visuomotor delay [31,32] the correction response was identified as the point at which the hand trajectory exceeded the ±1.5 SD threshold around the average non-perturbed response following 80ms post target perturbation [31]. The correction latency was determined as the time at which this corrective response was first identified following a target perturbation. If no corrective response was identified no response was recorded for that trial.

The maximum path offset (MPO) was also calculated for target perturbation trials. This provides a measure of how much the trajectory deviates from a straight line (i.e. non perturbed trials) and can provide insight into the intensity of the compensation in trajectory elicited by the correction [29]. Specifically, Atkeson and Hollerbach [29] proposed the path linearity measure as a means to quantify differences in the curvature of movement trajectories. Their measure of linearity or path offset is calculated as L = A/B where A is the maximum normal distance from the movement path to a straight-line joining start and end points and B is this straight-line distance (see Fig 1C). Essentially, if L = 0, the path is a perfect straight line; if L = 1, the path is circular. We take the maximum of these values as a measure describing the maximum deviation from linearity or MPO. The latency at which this MPO occurs provides us with an additional means of assessing the latency of the corrective response that does not rely on the comparison of trials in which a perturbation occurred with those in which a target perturbation does not. This is beneficial as a comparison of non-perturbed and perturbed target trajectories, while commonly utilised to estimate the correction latency, may over estimate this latency due to the use of an explicit threshold that must be exceeded in order to identify a response In this way [33], the MPO provides us with a measure of correction that is assessed independently from non-perturbation trials and is not affected by the limitations seen within the threshold method. Taken together, these measures allow us to gain an understanding of the functional output of the sensorimotor system as it relates to the online control of movement across temporal and spatial avenues.

We first assessed whether there was a directional effect based on direction of target perturbation on the dependent variables. While some previous work has demonstrated directional differences of extreme contralateral and ipsilateral reaching movements, performance differences have largely been quantified on a larger scale compared to what was used in this study, for example 10–20 degrees eccentricity [34] and up to 38 degrees eccentricity [35] Given the range of movement elicited within this experiment (±10°) is not considered far from the midline, is well within the binocular field of view (at this viewing distance, this is less than half the width of a keyboard) and previous work conducted within our lab has not identified an effect of direction over these smaller eccentricities [15] we did not hypothesize there to be a directional effect of perturbation direction. Indeed, it is common practice within similar areas of investigation [19, 36] to collapse across direction within these eccentricities. In the interest of being thorough however, and in line with Rossit and Harvey [13], we tested an effect of perturbation direction (left or right) for each of our dependent variables. Where the effect of perturbation direction was not statistically significant data were collapsed across perturbation direction. This was the case for dependent variables: reach latency, reach absolute accuracy and acceleration time. Where preliminary analysis determined there to be a significant effect of perturbation direction, this term was included in the statistical analysis. This was conducted for dependent variables: reach duration, deceleration time, peak velocity, correction latency, the MPO and the latency of MPO.

Dependent variables were analysed using a linear mixed model (LMM) procedure run in SPSS version 25 [37]. To analyse the effect of group and perturbation time we ran a LMM procedure with age group (older and younger), and target perturbation time (none, 0ms and 200ms) as fixed effects and participant as a random effect with the variance structure modelled as variance components. Where preliminary analysis indicated that there was an effect of perturbation direction, we investigated this separately, due to the structure of the conditions, using a LMM procedure with age group (older and younger), target perturbation time (0ms and 200ms) and perturbation direction (left and right) as fixed effects and participant as a random effect with the variance structure again modelled as variance components. We used Type III F tests to test the significance of the main effects and report the F test results. We report the results of post hoc pairwise comparisons only for significant main effects. This is consistent with other examples demonstrating the appropriate reporting of LMM analyses for repeated measures designs [38]. This analysis structure was used to test each of our dependent variables separately. Where a significant main effect was detected post hoc pairwise analysis was performed and a Bonferroni adjustment for multiple comparisons was used. At present, there is no generally agreed measure of effect size that can be calculated for linear mixed models (e.g. [39]). To give insight into the proportion of the variance explained by the fixed and random effects specified in our LMM analyses, we calculated a pseudo R^2^ measure first proposed by Nakagawa and Schielzeth [40] and further developed by Johnson [41]. R^2^ marginal (R^2^ GLMM (_m_)) describes the variance explained by the fixed effects relative to the expected variance of the dependent variable (values 0–1), while R^2^ conditional (R^2^ GLMM (_c_)) can be considered the variance explained by the fixed and the random effects relative to the expected variance of the dependent variable (values 0–1). This analysis was implemented in Jamovi under the GAMLj: General Analyses for the Linear Model in Jamovi package [42]. As a more direct indicator into the differences in performance differences across group, we also calculated the percent difference (%dif) between scores of younger and older participants, where a significant main effect of group was found. Percent difference was taken as the absolute value of the difference between the two scores on any given dependant variable, divided by the average of those two numbers, multiplied by 100 and rounded to the nearest whole number.

## Results

### Reach latency

**Fig 2:**
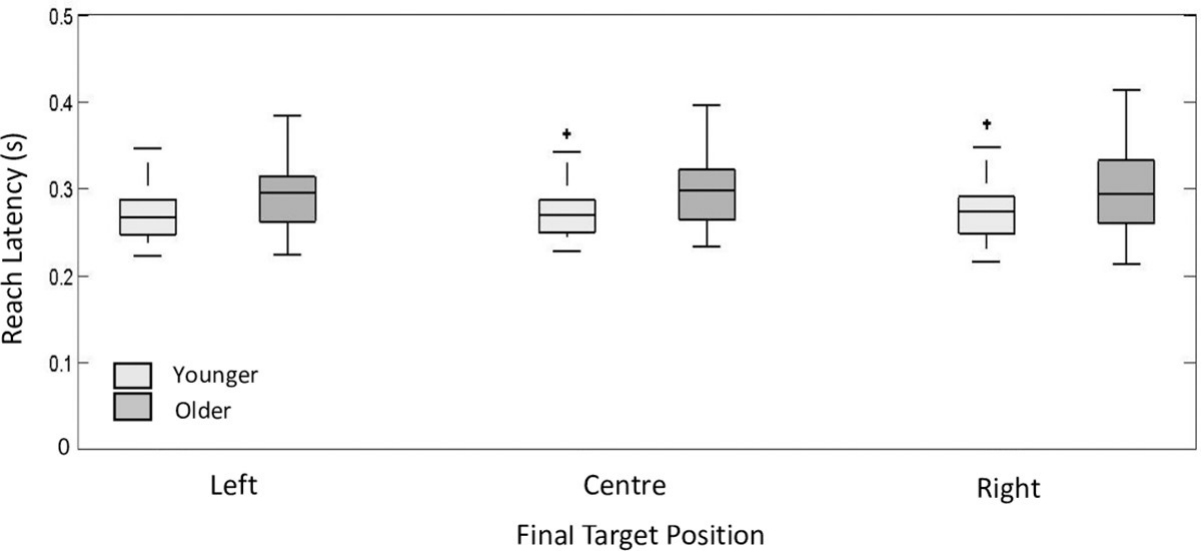
Reach latency. Box and whisker plots for reach latency for older and younger participants plotted as a function of perturbation time condition and target perturbation. The central mark is the median, the edges of the box are the 25th and 75th percentiles, the whiskers extend to the most extreme data points and the outliers are plotted individually and represented as +.

We first investigated whether there was a significant effect of age and perturbation time on reach latency. There was a significant group effect of reach duration (*F (*1, 34.13) = 4.51, *p =* 0.04). Older participants were shown to be consistently slower across all conditions with a mean difference of 28ms (10% percent difference) across all conditions (see Fig 2). Perturbation time did not have a significant effect on reach latency (*F (*2,140) = 1.91, *p =* 0.15) and the interaction between group and perturbation time was not significant (*F (*2,140) = 0.29, *p =* 0.75). This is unsurprising since the target perturbation occurred relative to the reach onset and therefore the perturbation time manipulation should not have had an effect on reach latency. Values for the R^2^ marginal and the R^2^ conditional measure for the dependent variable reach duration were R^2^_GLMM (m)_ = 0.10 and R^2^_GLMM (c)_ = 0.93. Older participants also had greater variability in their reach latencies as can be seen on the greater whisker length in Fig 2 and captured in the greater proportion of variance accounted by the R^2^ conditional measure.

### Reach duration

**Fig 3:**
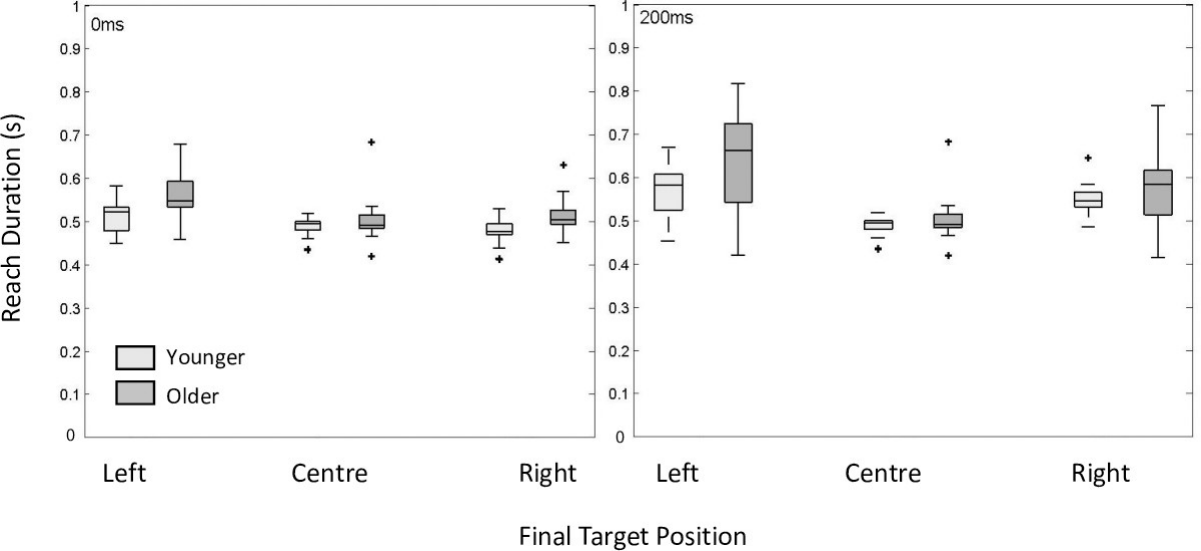
Reach duration. Box and whisker plots for reach duration for older and younger participants plotted as a function of perturbation time condition and target perturbation direction. Outliers are represented as +.

We next investigated whether there was a significant effect of age and perturbation time on reach duration. Despite the feedback designed to constrain the participant’s reach duration there was a significant group effect on reach duration (*F (*1, 35.61) = 5.59, *p =* 0.02). Older participants were consistently slower across all conditions with a mean difference of 36ms, or percent difference of 7%, pooled across conditions. Values for the R^2^ marginal and the R^2^ conditional measure for the dependent variable reach duration were R^2^_GLMM (m)_ = 0.35 and R^2^_GLMM (c)_ = 0.63. Older participants also had greater variability in their reach durations as in Fig 3 and captured in the greater proportion of variance accounted for by the R^2^ conditional measure.

There was a significant effect of perturbation time condition on reach duration (*F (*2,140) = 63.92, p < 0.001), post hoc pairwise analysis indicated a significant difference between the no perturbation condition and the 200ms condition (92ms) and the 0ms perturbation and the 200ms condition (72ms). There was no significant difference between the no perturbation condition and the 0ms perturbation condition (18ms). The interaction between group and perturbation time condition was not significant (*F (*2,140) = 1.86, *p =* 0.75) indicating these effects were stable across groups and perturbation conditions.

We further investigated the effect of perturbation direction on reach duration. There was a significant effect of perturbation direction (*F (*1,102) = 27.39, *p <* 0.001) on reach duration with the leftward perturbation resulting on average in a 39ms increase in reach duration (see Fig 3). There were no significant interactions between group and perturbation direction (*F (*1,102) = 2.95, *p =* 0.09), perturbation direction and perturbation time (*F (*1,102) = 0.01, *p =* 0.95) or group, perturbation direction and perturbation time (*F (*1,102) = 0.93, *p =* 0.34) suggesting that this effect was consistent across groups and perturbation conditions. The differences we see across group and perturbation condition in terms of reach duration are perhaps unsurprising given research investigating the speed accuracy trade of off [43]. As such it is important to consider reach duration in conjunction with the reach accuracy.

### Absolute accuracy

**Fig 4:**
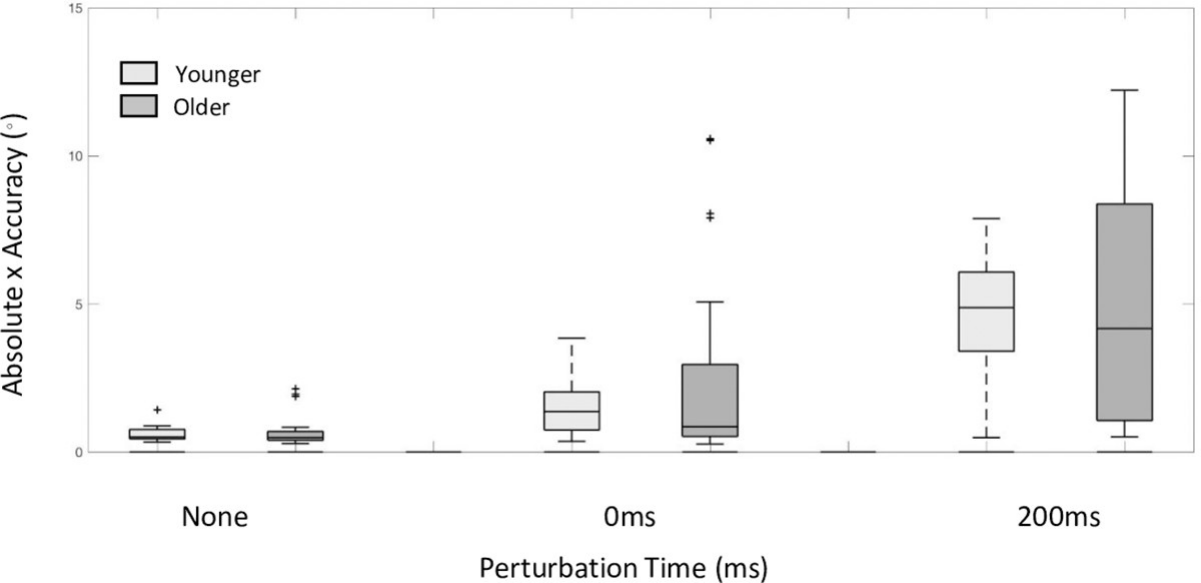
Absolute accuracy. Box and whisker plots for absolute x accuracy for older and younger participants plotted as a function of perturbation time condition and target perturbation. Outliers are represented as +.

Perhaps unsurprisingly, there was no significant group effect on reach absolute x accuracy (*F (*1, 35.13) = 0.165, *p =* 0.69). Fig 4 presents a box and whisker plots for absolute x accuracy for older and younger participants plotted as a function of perturbation time condition and target perturbation. It can be seen that the absolute touch accuracy for older and younger participants is on par. Furthermore, there is considerable variability in performance across participants particularly among the older group. Indeed, the values for the R^2^ marginal and the R^2^ conditional measure for the dependent variable absolute x accuracy were R^2^_GLMM (m)_ = 0.33 and R^2^_GLMM (c)_ = 0.69 indicating a significant proportion of the variance in performance is captured by individual participants.

The effect of perturbation time condition on absolute x accuracy was next considered. There was a significant main effect of perturbation time condition (*F (*2,140) = 95.25, p <0.001) with pairwise comparison indicating a significant difference across all perturbation times (none– 0ms = -1.22°, none– 200ms = -4.05° and 0ms-200ms = -2.82°). These results indicate that target perturbation resulted in decreased accuracy compared to stationary targets. This effect is exacerbated by the timing of the target perturbation with perturbations later in the reach additionally inaccurate. This is despite the increase in reach duration seen in the 200ms perturbation time condition, which according to Fitts’ Law [43] should have facilitated an increase in reaching accuracy.

### Acceleration time

**Fig 5:**
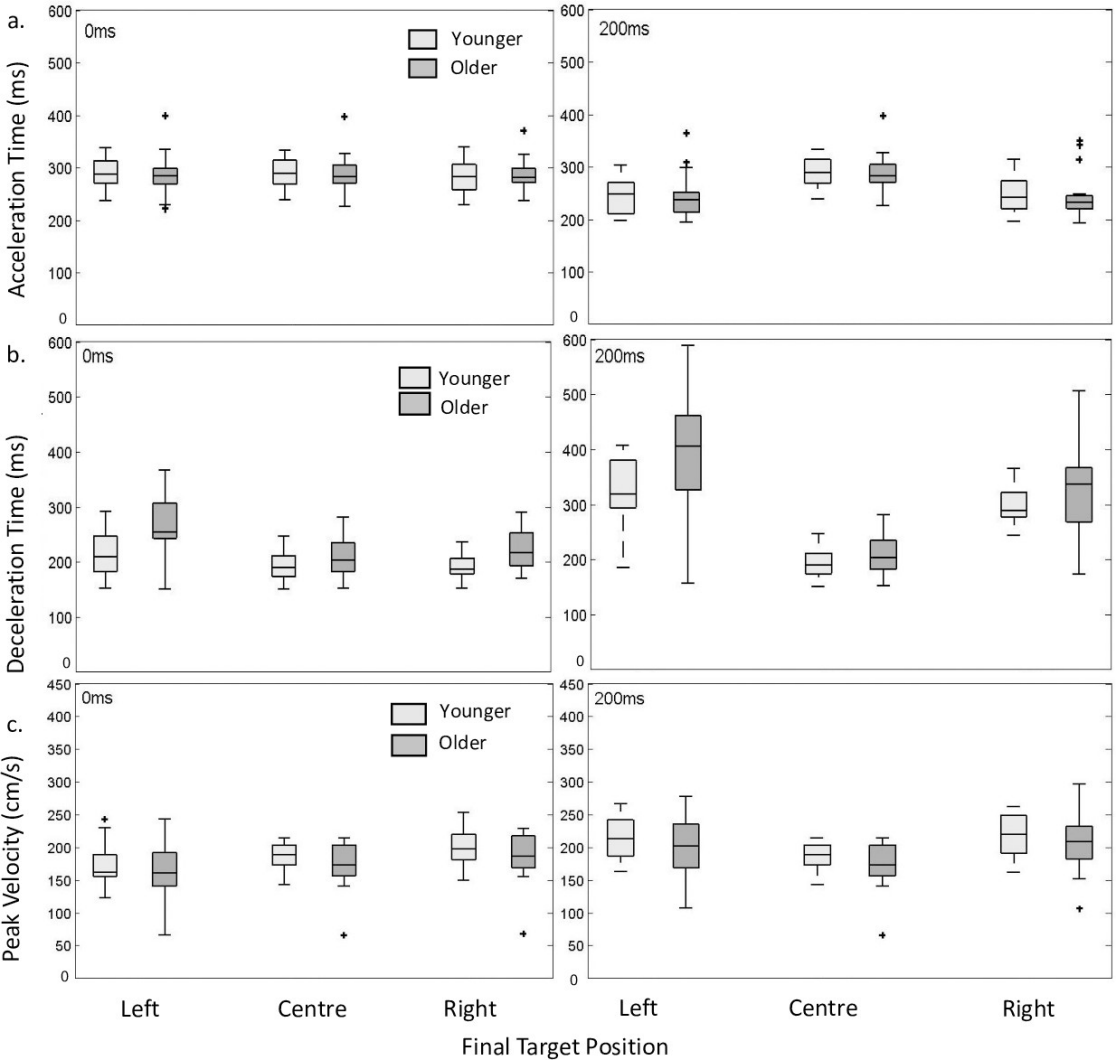
Acceleration time, deceleration time, peak velocity. Box and whisker plots for (a) acceleration time, (b) deceleration time and (c) peak velocity for older and younger participants plotted as a function of perturbation time condition and target perturbation. Outliers are represented as +.

To provide insight into the dynamics of the reach as it unfolds and in response to a target perturbation, we investigated the kinematic breakdown of the reaching moment. Differences in these kinematic markers across conditions give an indication into how the reach is recalibrated on the fly in response to the incorporation of new sensory information. We investigated whether there was a significant effect of age and perturbation time on acceleration time. There was not a significant group effect of acceleration time (*F (*1, 34.24) = 0.00, *p =* 0.95). However, perturbation time did have a significant effect on acceleration time (*F (*2,140) = 223.82, p <0.001). Post hoc pairwise analysis revealed that there was a significant difference between the no perturbation condition and the 200ms condition (mean difference = 45ms) and the 0ms and 200ms condition (mean difference (41ms). However, there was not a significant difference in acceleration time between the no perturbation condition and the 0ms condition (mean difference 3.77ms). As can be seen in Fig 5A both younger and older participants reached peak velocity earlier in the reach when the movement was associated with a perturbation 200ms after the reach onset but still after the initiation of the perturbation. The non-significant interaction between group and perturbation time (*F (*2,140) = 0.54, *p =* 0.95) supports this. Values for the R^2^ marginal and the R^2^ conditional measure for the dependent variable acceleration time were R^2^_GLMM (m)_ = 0.26 and R^2^_GLMM (c)_ = 0.90 indicating that there is a portion of variance uniquely contributed by participant differences.

### Deceleration time

We next investigated whether there was a significant effect of age and perturbation time on deceleration time. There was a significant group effect (*F* (1, 35.79) = 6.28, *p =* 0.02), older participants had an increased deceleration time across all conditions *(*37.10ms on average, a 15% difference). There was also a significant effect of perturbation time condition on deceleration time (*F (*2,140) = 140.53, p < 0.001) with post hoc pairwise analysis indicating a significant difference on average between the no perturbation condition and the 200ms condition (134.90ms) and the 0ms perturbation and the 200ms condition (113.08ms). There was no significant difference between the no perturbation condition and the 0ms perturbation condition (21.95ms). The interaction between group and perturbation time condition was not significant (*F (*2,140) = 0.54, *p =* 0.95) indicating these effects were stable across groups and perturbation conditions. Values for the R^2^ marginal and the R^2^ conditional measure for the dependent deceleration time were R^2^_GLMM (m)_ = 0.52 and R^2^_GLMM (c)_ = 0.71. This taken in consideration with inspection of Fig 5B demonstrates the variability in performance across participants and is particularly evident when inspecting the deceleration time of older participants in the 200ms target perturbation condition.

We further investigated the effect of perturbation direction on deceleration time. There was a significant effect of perturbation direction (*F (*1,102) = 24.53, p <0.001) on deceleration time with the leftward perturbation resulting on average in a 37.79ms increase in deceleration time. Inspection of Fig 5B supports this effect. There were no significant interactions between group and perturbation direction (*F (*1,102) = 3.62, *p =* 0.06), perturbation direction and perturbation time (*F (*1, 102) = 0.16, *p =* 0.69) or group, perturbation direction and perturbation time (*F (*1,102) = 0.29, *p =* 0.59) suggesting that this effect was consistent across groups, perturbation conditions and perturbation direction.

### Peak velocity

We next investigated whether there was a significant effect of age and perturbation time on peak velocity (Fig 5C). There was no significant group effect (1, 34.52) = 0.84, *p =* 0.36). Indicating that the peak velocity for both younger and older participants were comparable. However, there was a significant effect of perturbation time condition on peak velocity (*F (*2,140) = 54.79, p < 0.001). Post hoc pairwise analysis indicated that there was a significant difference between the no perturbation condition and the 200ms condition (29.84 cm/s) and the 0ms perturbation and the 200ms condition (30.54 cm/s). There were no other significant differences. The interaction between group and perturbation time condition was not significant (*F (*2,140) = 0.05, *p =* 0.95) indicating these effects were stable across groups and perturbation conditions. Values for the R^2^ marginal and the R^2^ conditional measure for the dependent peak velocity were R^2^_GLMM (m)_ = 0.16 and R^2^_GLMM (c)_ = 0.77.

We further investigated the effect of perturbation direction on peak velocity. There was a significant effect of perturbation direction (*F (*1,102) = 19.9, p < 0.001) on peak velocity with the leftward perturbation resulting on average in a 13.97 cm/s increase in peak velocity. There were no significant interactions between group and perturbation direction (*F (*1,102) = 0.11, *p =* 0.918), or group, perturbation direction and perturbation time (*F (*1,102) = 0.288, *p =* 0.592). There was a significant interaction between perturbation direction and perturbation time (*F (*1,102) = 11.38, < = 0.001).

### Correction latency

We next investigated whether there was a significant effect of age and perturbation time on correction latency. There was no significant group effect (*F (*1, 33.15) = 3.11, *p =* 0.87), indicating that correction latencies for both younger and older participants were comparable. It is interesting to note that although correction latencies did not differ between age groups, older participants performed a correction much less often that their younger comparisons (see Table 1), this is also supported by the significant association between age group and correction performance χ(1) = 85.77, p = 000.

**Table 1:** Percentage of trials which elicited a correction in response to a target perturbation.

Group	0ms Left	0ms Right	200ms Left	200ms Right
Younger	100	100	72	66
Older	91	92	59	48

**Fig 6:**
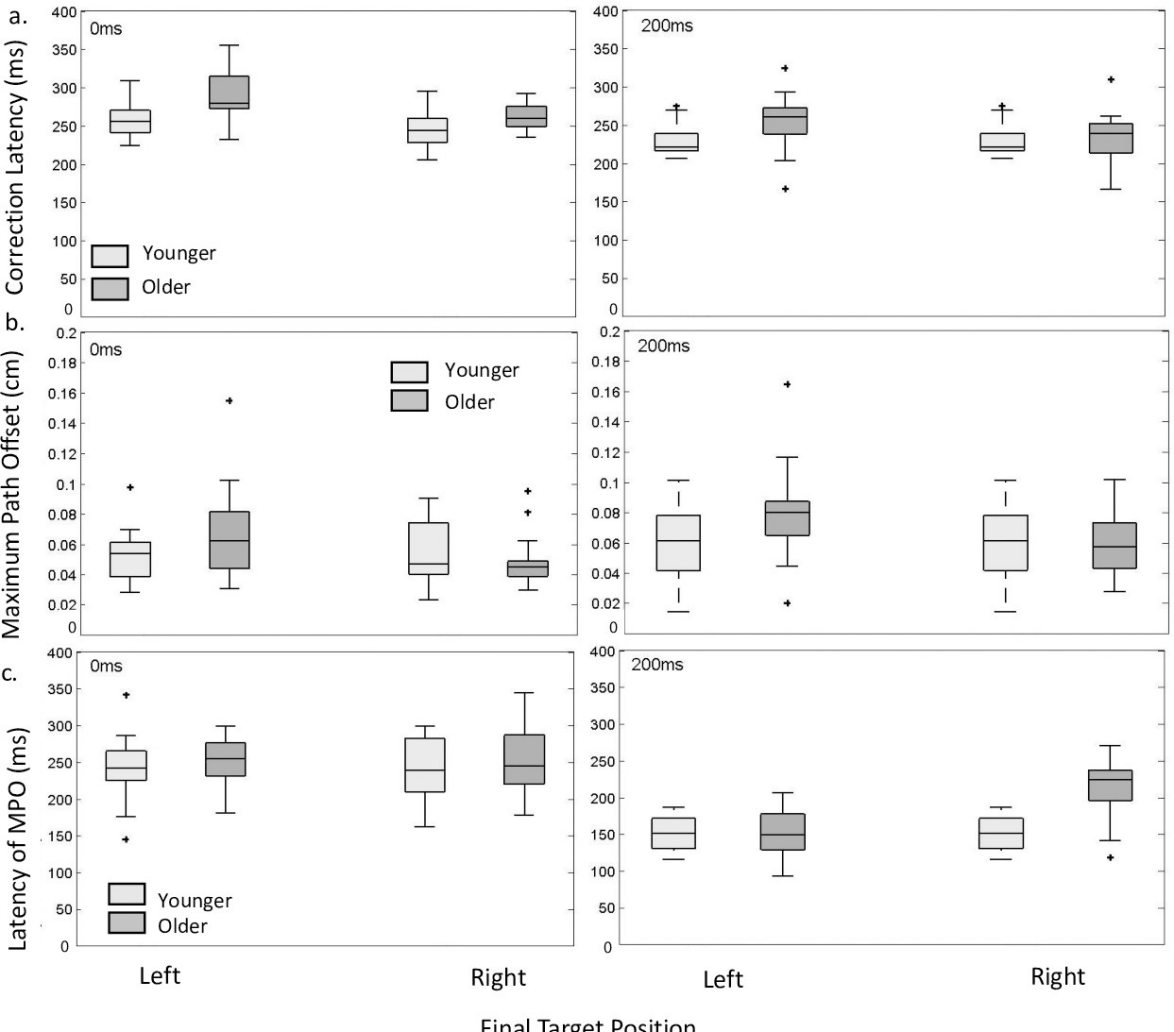
Correction latency maximum path offset and latency of MPO. Box and whisker plots for (a) Correction latency, (b) Maximum Path Offset and (c) Latency of MPO for older and younger participants plotted as a function of perturbation time condition and target perturbation direction. Outliers are represented as +.

There was a significant effect of perturbation time condition on correction latency (*F (*1,98.34) = 49.48, *p =* 0.00) with post hoc pairwise analysis indicating a significant difference between perturbation conditions with the 200ms condition producing an on average 32.68ms faster correction latency compared to the 0ms condition (see Fig 6A). There was also considerable variability in correction latency across participants. This variability in participant’s performance is further evidenced through the additional variance explained by the R^2^ conditional measure beyond that of the R^2^ marginal. Values were R^2^_GLMM (m)_ = 0.25 and R^2^_GLMM (c)_ = 0.55.

There was also a main effect of perturbation direction on correction latency (*F (*1,98.34) = 8.62, *p =* 0.04) with target perturbations to the right producing, on average, a 13ms faster correction compared to leftward target perturbations. There was no significant interaction between group and perturbation time (*F (*1, 98.34) = 0.167, *p =* 0.68) nor was there a significant interaction between group and perturbation direction (*F (*1, 98.34) = 0.16, *p =* 0.69). The interactions between perturbation time (*F (*1, 98.34) = 0.64, *p =* 0.80) and perturbation and group, perturbation and perturbation direction (*F (*1, 98.32) = 0.14, *p =* 0.71) were not significant.

**Fig 7:**
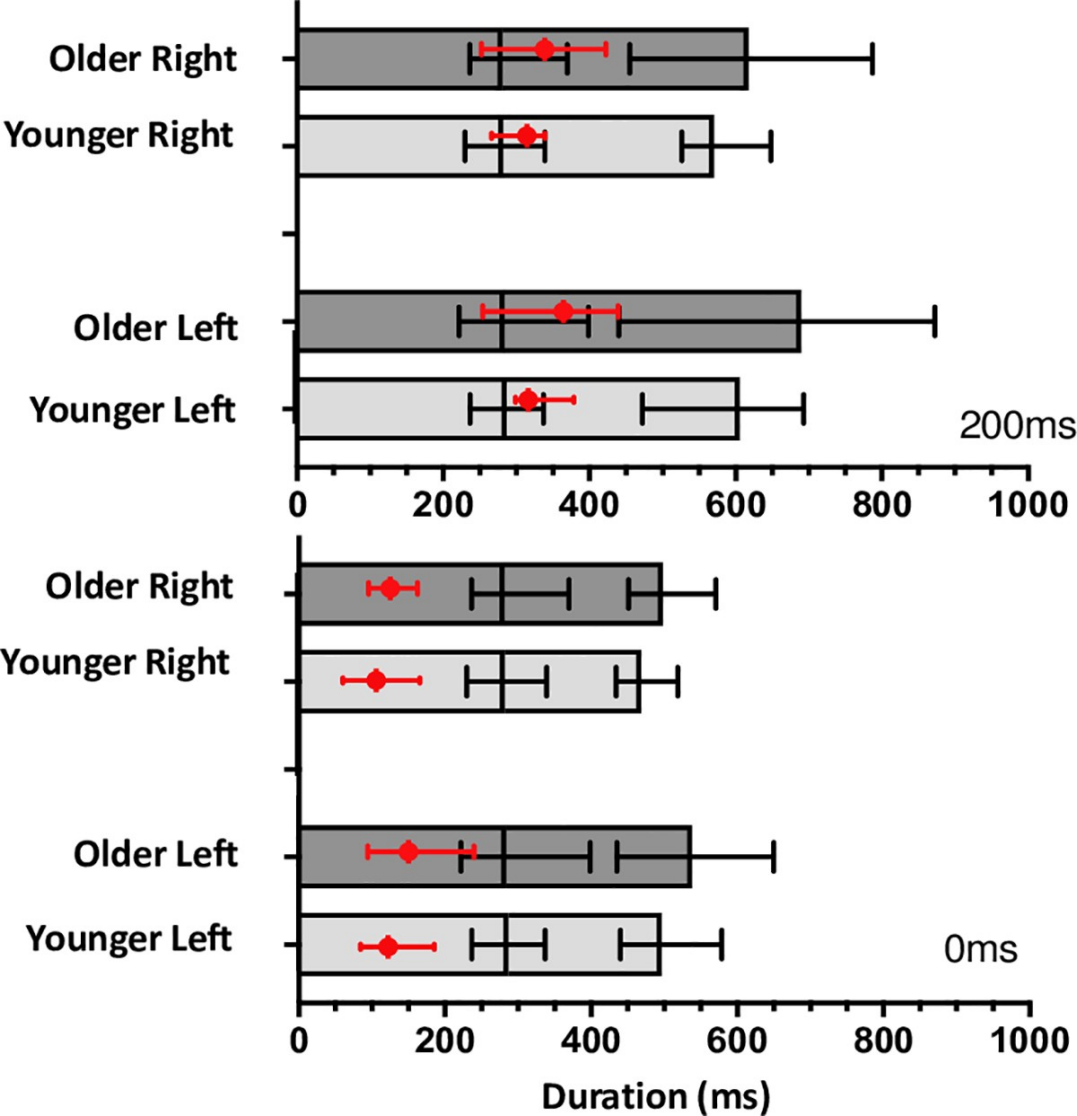
Stacked acceleration and deceleration time plots. Measures presented as a function of group and target perturbation time overlaid with correction latency. Acceleration time is presented in the first section of the stacked bar plots. Mean acceleration time is indicated by the first vertical black line. Horizontal error bars for mean acceleration time are presented in black and represent the minimum and maximum values. Deceleration time is presented in the second section of the stacked bar plots. Mean deceleration time is indicated by the end of the second vertical line. Horizontal error bars for mean deceleration time are similarly presented in black and indicate the minimum and maximum values. Mean correction latency represented in red is overlaid with horizontal error bars denoting the minimum and maximum values.

As presented above, our results indicate that trajectory corrections initiated to perturbations that occur later in the reach are initiated faster than those that occur in response to perturbations earlier in the reach. To provide more insight into this effect and understand the dynamics of the correction latency relative to the kinematic breakdown of the reach, we plotted the correction latency relative to the acceleration and deceleration time. This provides a visual description of how the correction latency interacts with the reach phase in which it occurs. Fig 7 shows a stacked acceleration time and deceleration time plot as a function of both group and final target position. Acceleration time is presented in the first section of the stacked bar plots and the mean is denoted by the vertical black line. Horizontal error bars for mean acceleration time are presented in black and represent the minimum and maximum value. Deceleration time is presented in the second section of the stacked bar plots with the mean indicated by the end of the bar and the mean values is indicated by the final edge of the filled box. Horizontal error bars for mean deceleration time are similarly presented in black and indicate the minimum and maximum values. The stacked bar plots are overlaid with the mean correction latency represented in red with horizontal error bars denoting the minimum and maximum values. It is clear that corrective movements that occur in response to later target perturbations largely occur within the deceleration phase of the reach whereas responses to target perturbations that occur at reach onset are produced during the acceleration phase of the reach. The differences in reach phase in which the correction is elicited may help explain the differences in correction latencies we see across the perturbation time conditions.

### Maximum path offset (MPO)

We next investigated whether there was a significant effect of age and perturbation time on MPO. Fig 6B presents the MPO as a function of group and perturbation time condition. There was no significant group effect (*F (*1, 33.15) = 2.67, *p =* 0.111), indicating that the MPO for both younger and older participants were comparable. However, there was a significant effect of perturbation time condition on MPO (*F (*1, 94.22) = 11.047, *p =* 0.001) with post hoc pairwise analysis indicating a significant difference between perturbation time conditions 0ms and 200ms (mean difference = -0.01 cm). There was also a main effect of perturbation direction on MPO (*F (*1, 92.57) = 20.58, p <0.001) with post hoc pairwise analysis indicating a significant difference between directions right and left (mean difference = -0.013 cm). There was no significant interaction between group and perturbation time (*F (*1, 94.22) = 3.73, *p =* 0.06) but there was a significant interaction between group and perturbation direction (*F (*1, 92.57) = 10.69, *p =* 00). The interactions between perturbation time and perturbation direction and the interaction between group, perturbation time and perturbation direction were not significant (*F (*1, 92.57) = 1.07, *p =* 0.30 and *F (*1, 92.57) = 0.31, *p =* 0.58, respectively). Values for the R^2^ marginal and the R^2^ conditional measure for the dependent deceleration time were R^2^_GLMM (m)_ = 0.20 and R^2^_GLMM (c)_ = 0.54.

### Latency of maximum path offset

In conjunction with the MPO we investigated the latency of the MPO to provide a separate insight into the timing of the corrective response in addition to correction latency. We next investigated whether there was a significant effect of age and perturbation time on the latency of the MPO. Fig 6C shows the latency of the MPO as a function of group and perturbation time condition. There was no difference in latency of the MPO across younger and older participants (*F (*1, 31.70) = 2.62, *p =* 0.115). However, there was a significant effect of perturbation time condition on MPO (*F (*1,94.64) = 124.51, p <0.001) with post hoc pairwise analysis indicating a significant difference between perturbation time conditions 0ms and 200ms condition, with the later perturbation time condition eliciting a correction on average 70.95ms faster than the 0ms condition. There was also a main effect of perturbation direction on the latency of MPO (*F (*1, 92.21) = 7.52, *p =* 0.007) with post hoc pairwise analysis indicating a significant difference between directions right and left (mean difference = 17.37ms). There was no significant interaction between group and perturbation time (*F (*1, 94.64) = 1.36, *p =* 0.25) nor was there a significant interaction between group and perturbation direction (*F (*1, 92.21) = 2.47, *p =* 0.12) but there was a significant interaction between perturbation time and perturbation direction (*F (*1, 92.21) = 10.54, *p =* 0.00). There were no other significant interactions. Values for the R^2^ marginal and the R^2^ conditional measure for the dependent deceleration time were R^2^_GLMM (m)_ = 0.50 and R^2^_GLMM (c)_ = 0.58.

## Discussion

The aim of the current study was to quantify how the online control of voluntary reaching is modulated by ageing. We used an analysis of the latency and accuracy of reach trajectory corrections to investigate double-step reaching under time pressure to perturbations at different times during the reach. Perturbations that occurred early in the reach (i.e. 0ms) were more easily accounted for than those that occurred later in the reach (i.e. 200ms post reach initiation). This was consistent across both younger and older participants. Corrections to target perturbations that occurred later in the reach were also elicited faster for both younger and older participants compared to perturbations that occurred at reach onset, potentially due to the time in the reach in which the correction is produced. However, difficulties in compensating for target perturbations that occurred later in the reach were exacerbated in older participants who produced a correction proportionally less often compared to their younger counterparts and had increased deceleration times.

Despite explicit feedback designed to constrain the reach duration, older participants were consistently slower than younger participants. This is perhaps unsurprising given the evidence that older participants are both slower to initiate and execute a goal directed reach. The age-related increase in reach duration was persistent across perturbation time conditions, suggesting older participants were slower overall. However, perturbations that occurred later in the reach, i.e. the 200ms perturbation time condition, resulted in a significantly larger increase in reach duration for both younger and older participants when compared to the 0ms and no target perturbation conditions (see Fig 3). Furthermore, there was no significant difference between the no target perturbation condition and the 0ms target perturbation. This is in line with a large body of evidence suggesting that responses to target perturbations can be accounted for without a significant increase in reach duration, though these findings are largely based on perturbations that occur within the early stages of the reaching movement (for a review see [44]). It is also interesting to note that there was a group effect of age persisted despite consistent feedback regarding reach duration.

Indeed, performance differences across perturbation time conditions are particularly informative when the reach duration is considered alongside the accuracy of the touch. Touch accuracy responses to perturbations that occurred later in the reach (200ms) were significantly less accurate compared to the early perturbation condition (0ms) and the no perturbation condition (see Fig 4). This suggests that increases in reach duration did not afford a faciliatory effect on touch accuracy. Furthermore, there was a significant difference between the 0ms perturbation condition and the no perturbation condition, indicating that despite the time at which a perturbation occurs, difficulty in compensating for it results in an increase in reach duration and decrease in touch accuracy. This is also exacerbated by the time at which the perturbation occurs, particularly with older participants. Liu and Todorov [2] similarly found incomplete corrections to target perturbations that occurred 300ms after reach onset, despite providing additional time to complete the movement (total movement time was 600-800ms across a 30cm reach). They suggest a potential mechanism in for this effect. Specifically, achieving stability in stopping a reaching movement may potentially compromise the sensorimotor system’s ability to respond to positional errors at the end of the reach. We speculate that target perturbations that occur later in the reach increase the task difficulty. As Teeken et al [45] suggested, differences in age related performance become particularly apparent when the difficulty of the task is increased. Given the later target perturbations occurred considerably closer to the end of the reaching movement than the early target perturbations, these may be quantitatively and qualitatively more difficult to compensate for and therefore we observed a greater effect of age. This increased difficulty is presumably due to the minimum amount of time needed in order to incorporate new sensory information into an ongoing movement [3, 15] or a compromised ability to detect positional errors in the later stages of the movement [2] and is evident in the increased duration and decreased accuracy seen in our results.

There was also a wide spread of reach duration and touch accuracy scores (see Figs 4 and 5). While this is evident to some extent across both younger and older participants, the older group produced a much wider range of performance scores, especially within the 200ms perturbation time condition for both reach duration and touch accuracy. This trend of greater variability for older people for reach duration and touch accuracy is evident in the additional variance explained by R^2^ conditional compared to R^2^ marginal. Interestingly, there was a significant effect of perturbation side for reach duration and the spread of these performance scores can be seen within the left direction perturbation in the 200ms perturbation condition. We found it curious that there was an effect of perturbation direction. As stated briefly within the methods section, some previous work has demonstrated directional differences of extreme contralateral and ipsilateral reaching movements [34, 35]. Differences in performance however, have largely been quantified on a much wider scale compared to what was is used in this study, for example 10–20 degrees eccentricity [34] and up to 38 degrees eccentricity [35]. Given the range of movement elicited within this experiment (±10°), at this viewing distance, this is less than half the width of a keyboard and previous work conducted within our lab has not identified an effect of direction over these smaller eccentricities [15] we did not hypothesize there to be a directional effect of perturbation direction. While it is curious that we see an effect of direction, the large variability in performance we see within the older participants indicates that this effect should be interpreted with caution. Given its significance however, perhaps this is a question for future research.

The focus of this discussion thus far has been largely centred on outcome measures of the reaching movement that provide insight into the functional output of the system to enact its goal. The kinematic breakdown of the reaching movement provides insight into the dynamics of the reach with the ability to detect subtle changes in kinematic performance variables that are sensitive to the online control process. While there was no difference between groups in terms of acceleration time, there was a group difference between deceleration times. The deceleration portion of the reach has been previously associated with explicit feedback-based control as this portion of the reach is used to ‘home in’ on the target position and facilitate accuracy [46]. One hypothesis for this is that older participants may rely more heavily on explicit visual feedback of the target and hand positions in the later stages of the reach to compensate for any age-related effects of performance in movement execution [47]. Interestingly, there was no group difference between the peak velocities of older and younger participants indicating that in terms of maximum speed the performance between groups was comparable. This suggests that the increase in reach duration seen within the older participants is largely associated with an increase in deceleration time that is consistent with previous findings [48];

To investigate the corrective movement elicited in response to a target perturbation we used a combination of measures designed to provide insight into not only the latency of the correction but the magnitude of the corrective response. Our results indicate that there were no differences between the older and younger groups in terms of the latency of the corrective response, as described by the correction latency and the latency of the MPO, suggesting that the corrective response to the perturbation across both age groups were elicited with comparable latency. This is interesting as previous work has demonstrated that older participants were able to initiate a correction in response to an early target perturbation [10]. This information combined with the evidence that older participants typically spend an increased portion of their reach in the deceleration phase, suggests that there may be some age specific related difficulties in compensating for the target perturbation. However, if we consider the proportion of corrections elicited in response to a target perturbation, we can see that overall older participants produced a correction for substantially less perturbation trials compared to younger participants (see Table 1). This is consistent with previous work by Sarlegna [10] that investigated the capacity for older participants to successfully and accurately account for a target perturbation. Although there are differences in quantification, Sarlegna [10] similarly found that older participants accounted for proportionally less of the target perturbation than younger participants. Taken together, it can be seen that while older participants may elicit a correction to a perturbation with a similar latency to that of younger participants, they do so less frequently.

There was a significant effect of perturbation time condition, with target perturbations that occurred later in the reach (200ms) producing faster correction latencies than those that occurred earlier (0ms). This pattern of results is consistent across both temporal measures describing the corrective response, correction latency and latency of MPO. This pattern of results is somewhat surprising given the incomplete nature of responses to target perturbations that occurred later in the reach, that is a decrease in both the correction frequency and overall accuracy. Incomplete updating for target perturbations that occur later in the reach has been demonstrated by a number of studies investigating disruptions to task requirements throughout the reach such as Liu and Todorov [2] and previous work by our group [18]. Given the research detailing a consistent sensorimotor delay when incorporating feedback into an ongoing movement [49] this was perhaps a bit surprising, especially considering the direction of effect and the overall performance across other measures in this condition. However, when we consider the phase of the reach in which these target perturbations occur, this can provide us with some insight to help explain the potential mechanisms. Fig 7 provides a kinematic breakdown of reach duration partitioned into acceleration and deceleration time with the average correction latency overlaid. It can be easily seen that corrections in response to target perturbations that occur at reach initiation, i.e. the 0ms condition, occur before the movement has reached peak velocity, within the acceleration time, while responses to perturbations which occur later in the reach, the 200ms, condition occur largely within the deceleration phase of the movement. The differences in phase of the reach in which the correction is elicited may help explain the differences in correction latencies we see across the perturbation time conditions. While adjustments to the ongoing movement plan have been known to occur at any stage of the reach, the deceleration phase of the movement is associated with explicit feedback induced corrective responses to improve reaching accuracy. It is also possible that changes to reaching movements that occurred after peak velocity were more easily implemented, perhaps due to the decreasing speed of the movement. While participants, particularly older participants, tended to correct proportionally less and with decreased accuracy for later perturbation time conditions they do so with a faster latency than in the 0ms condition.

The results of this study provide insight into changes to online control related to healthy ageing. While many previous studies investigating the age-related changes to online control performance have perturbed targets only at movement initiation, in this study we investigated the effect of target perturbations that occurred at different times throughout the entire reach. Our evidence suggests that certain aspects of online sensorimotor integration are compromised within older participants. As discussed above, the differences were especially prominent at certain times in the reach. The delayed movement times are consistent with previous work, as is our finding of accurate reaches under the no update condition. These results may be a by-product of overall slowing of movement speed with age, or changes in strategy for online updating with age. More work is needed to tease apart these alternatives.

Changes to sensorimotor control with healthy ageing could be attributed to multiple factors, and therefore the underlying neural mechanisms are not yet clear. Previous studies have suggested age-related effects on motor performance may be due to a variety of factors including but not limited to; increased noise (sensory, motor, neural or a combination), within the sensorimotor system [50], degradation in aspects of vision and visual performance [6], deficits in movement planning and movement execution [47] performance deficits in speed of processing [51], neurological changes to the brain architecture [52] and strategic manipulations of performance [53]. While it is the case that the potential mechanisms noted above span across multiple and very different levels of explanation (e.g. behavioural / sensory / physiological) it is likely that these mechanisms are not mutually exclusive and, on some levels, may exist simultaneously and in combination to produce the age-related effects we see on performance. Our results are not inconsistent with any of these potential mechanisms though at present, no model of sensorimotor behaviour formally accounts for age-related changes to performance in a quantifiable and testable way. One of the first steps in developing a model of sensorimotor control that is valid across age is to provide an account of online control and ageing across a controlled parameter space. This will help elucidate some of the key changes in performance as we age, which coupled with models of sensorimotor control that consider age related changes in performance can be mapped back onto potential underlying mechanisms.

## Conclusion

The aim of this study was to understand how ageing affects online control at different times in the reach. Our goal was to quantify the latency and error of the corrective response to a target perturbation at different times in a reach, across age groups. Perturbations that occurred early in the reach (i.e. at reach initiation) were more easily accounted for in terms of accuracy and overall reach duration than those that occur later in the reach (i.e. 200ms post reach initiation). This is despite later target perturbations eliciting a faster correction latency than those that occurred earlier in the reach. We speculate that the time in the reach at which the correction is elicited help explain the differences in correction latencies observed across the perturbation time conditions. The difficulties in compensating for target perturbations that occurred later in the reach were exacerbated in older participants and resulted in longer deceleration times. While there was no group difference in terms of accuracy, older participants were considerably slower overall and elicited a correction proportionally less than younger participants despite statistically equivalent correction latencies. This pattern of results indicates that older participants are less likely to initiate late corrections but are able to correct when they do choose to, suggesting that ageing impacts the online control of goal directed movements.
